# Supplementary material for: Intratumoral modulation of the inducible co-stimulator ICOS by recombinant oncolytic virus promotes systemic anti-tumour immunity
Source: Nat Commun. 2017 Feb 13;8:14340. doi: 10.1038/ncomms14340 (PMC5316835; doi:10.1038/ncomms14340)
Supplement: Supplementary Information — Supplementary Figures and Supplementary Tables [file ncomms14340-s1.pdf]

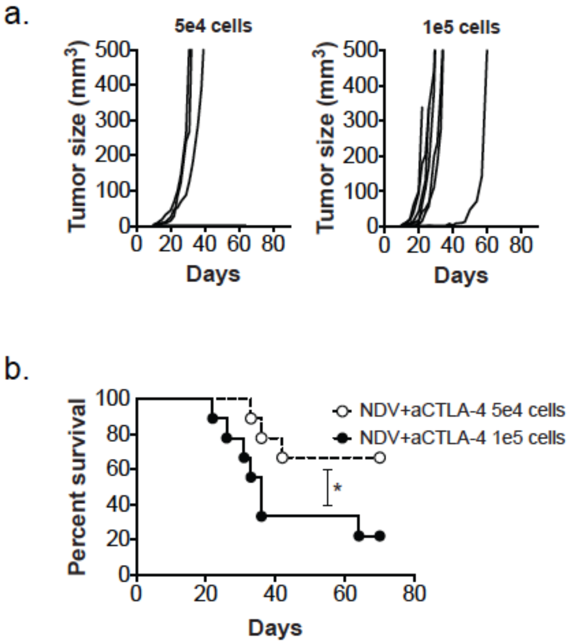

Supplementary Figure 1. Therapeutic efficacy of combined NDV and anti-CTLA-4 therapy is attenuated with a larger tumor challenge. Bilateral flank B16-F10 tumors were established as described and the animals were treated with 4 intratumoral injections to the right tumor. a) Growth of distant tumors. b). Overall survival. *$p<0.05$. Pooled data from 2 experiments with 5 animals per group.

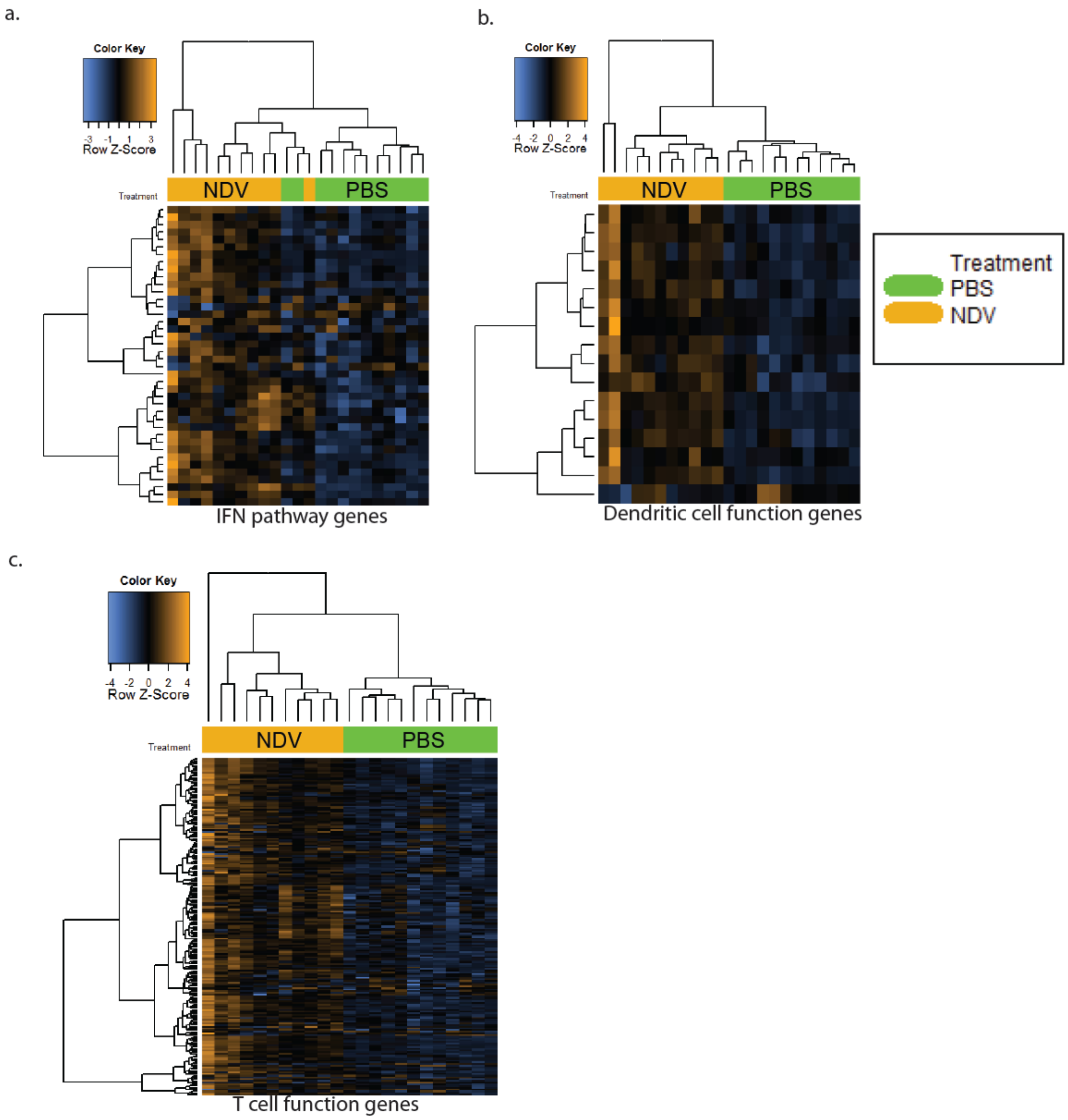

Supplementary Figure 2. Upregulation of immune-related genes in response to NDV. RNA isolated from NDV-infected tumors was subjected to gene expression analysis using the Nanostring Mouse Pancancer Immune Profiling panel. a) Type I interferon pathway genes. b) Dendritic cell function genes. c) T cell function genes. The actual genes, level of induction, and statistical significance are outlined in Supplementary Tables 1-3. Representative data from 2 experiments with 10 animals per group.

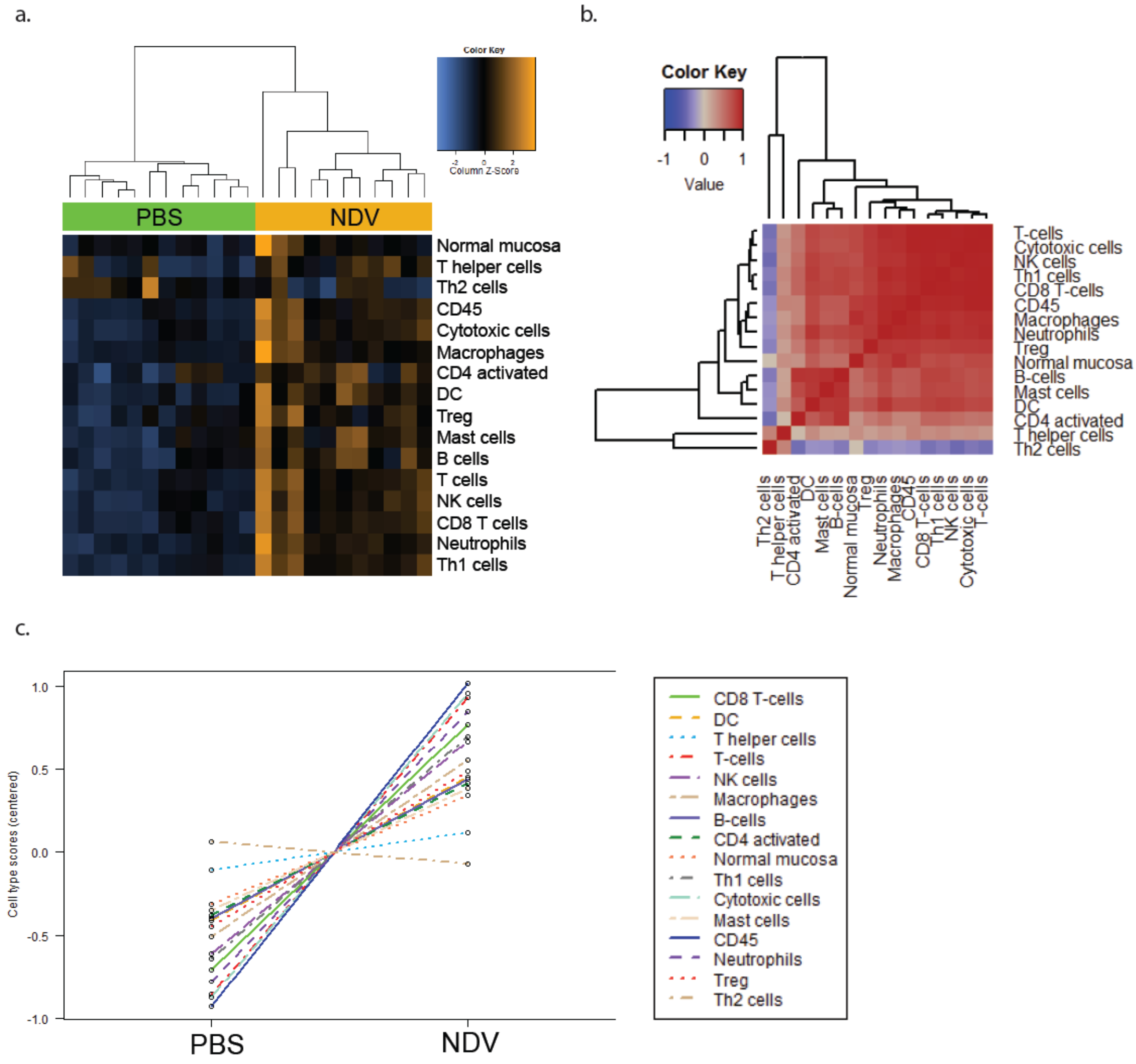

Supplementary Figure 3. NDV infection induces immune transcriptional signatures defining Th1 and CTL, but not Th2 responses. RNA isolated from NDV-infected tumors was subjected to gene expression analysis using the Nanostring Mouse Pancancer Immune Profiling panel. Immune cell subtypes were defined based on the transcriptional signatures developed by Bindea et al. (*48*). a) Heatmap showing raw abundance of immune cell types in each sample, compared to other samples. Orange indicates high abundance; blue indicates low abundance. b) Heatmap showing the correlation matrix of raw immune cell abundance. Red and blue indicate positive and negative correlation, respectively. c) Covariate plot of raw cell type abundance measurements of treated vs. control samples. Representative data from 2 experiments with 10 animals per group.

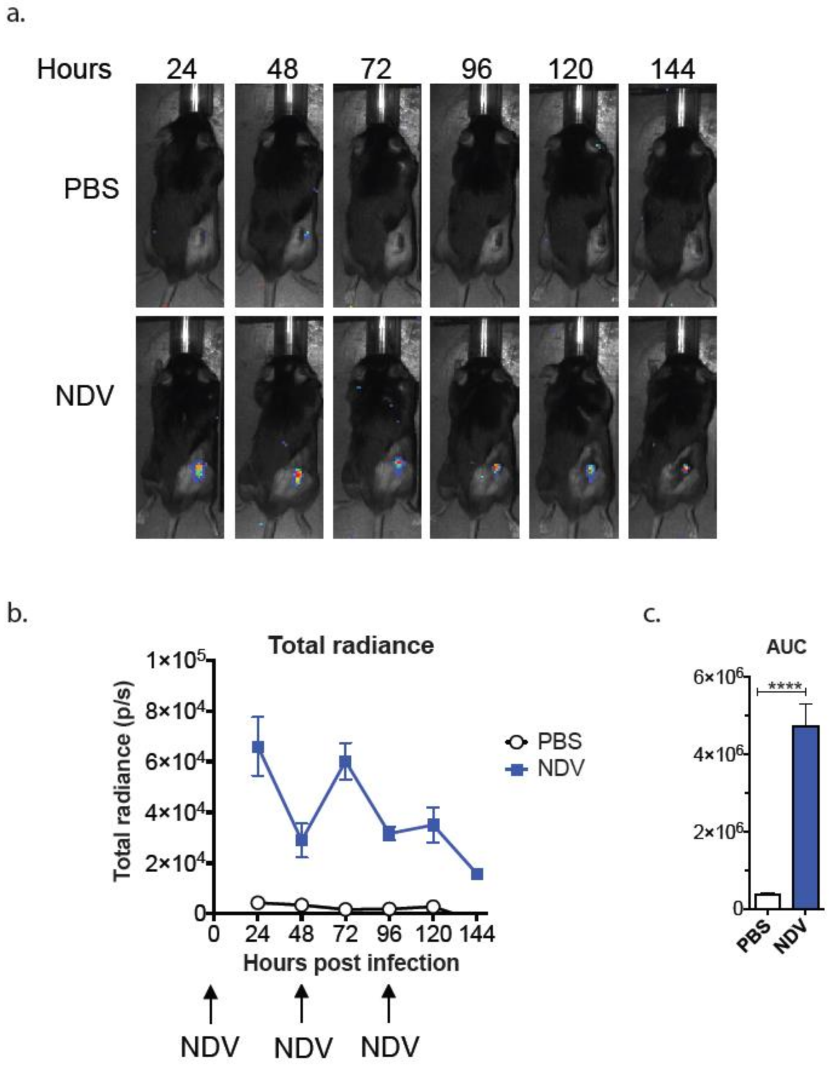

Supplementary Figure 4. Expression of transgene from NDV *in vivo*. Animals bearing flank B16-F10 tumors were injected intratumorally with NDV expressing firefly luciferase (NDV-fluc) on the indicated days and analyzed on IVIS Imaging System. a) Representative luminescence images. b) Quantified total radiance from tumor area in photons per second (p/s). c) Area under the curve (AUC) calculated from (b). Representative data from 2 experiments with 5 animals per group. Data with error bars represent mean +/- SEM. ****p<0.0001.

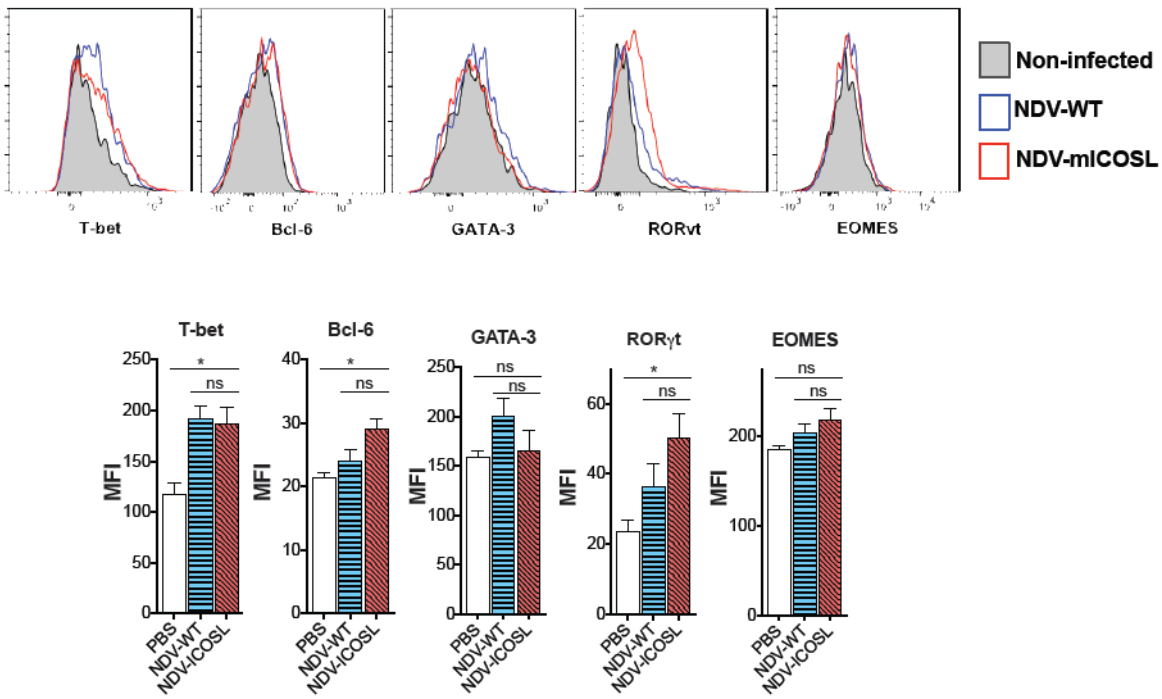

Supplementary Figure 5. Expression of lineage-specific transcription factors in the CD4+FoxP3- tumor lymphocyte subsets. Top: flow cytometry plots gated on the CD4+FoxP3- (Tcon) TIL population. Bottom: Median fluorescence intensity values calculated from the plots above with average of 5 animals per group. *p<0.05, ns: non-significant. Data with error bars represent mean +/- SEM.

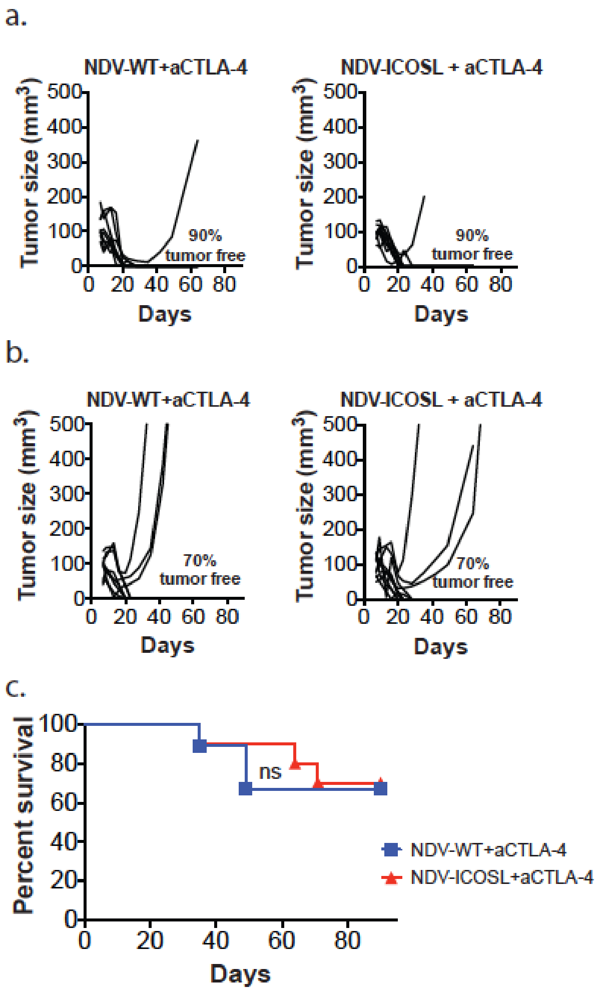

Supplementary Figure 6. Efficacy of NDV-ICOSL in non-NDV-permissive tumor cell line.

MB49 tumor cells were titrated to establish a model that resulted in 100% tumor take and 0% survival in untreated animals. Animals bearing bilateral flank MB49 tumors were treated intratumorally into the right tumor with the indicated NDV and intraperitoneally with anti-CTLA-4 antibody. a) Growth of individual right (virus-injected) tumors. b) Growth of distant tumors. c) Overall survival. ns: non-significant. Pooled data from 2 experiments with 5-10 animals per group.

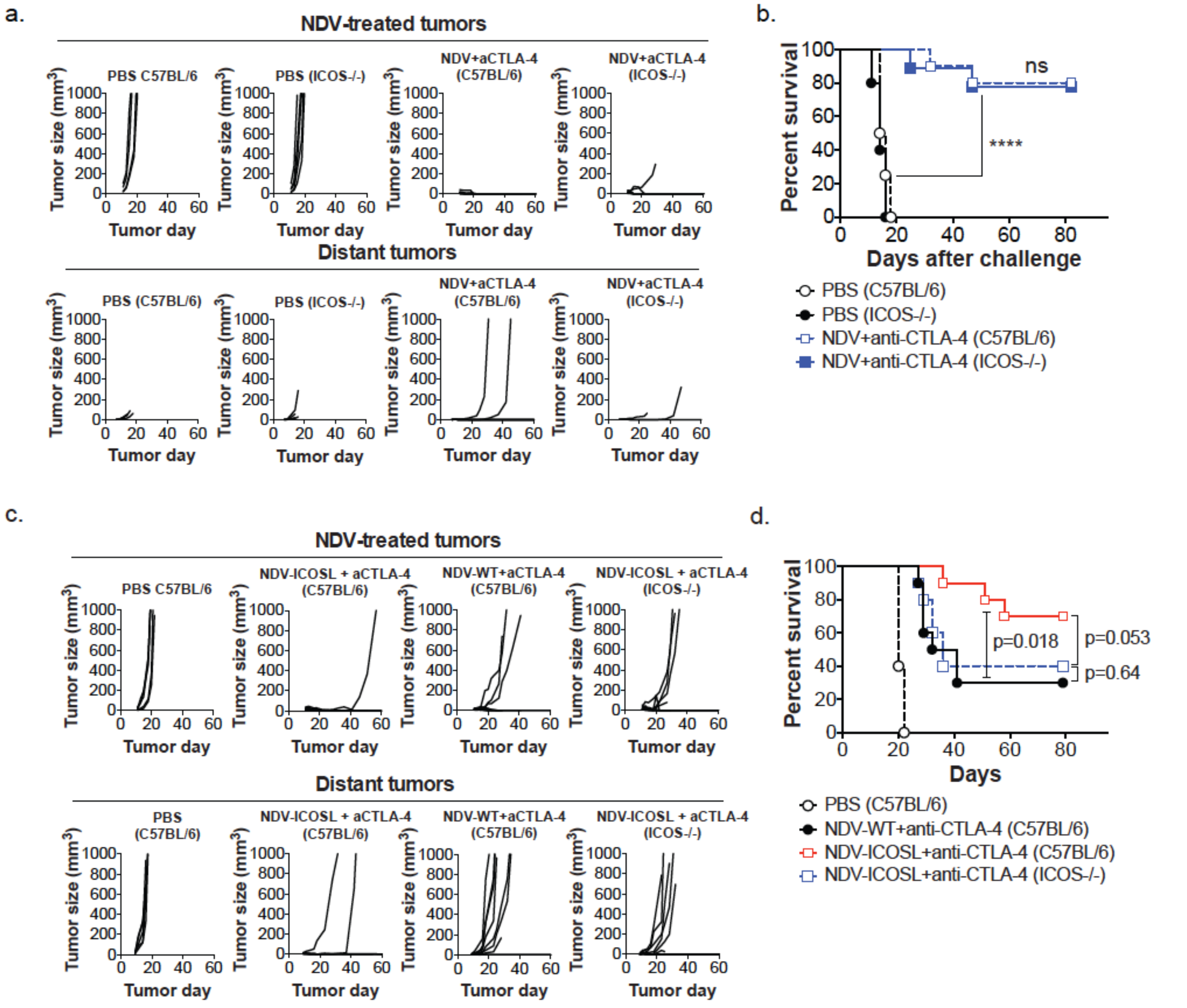

Supplementary Figure 7. Efficacy of NDV in ICOS-deficient mice. C57BL/6 ICOS-/- animals or sex and age-matched C57BL/6 controls were challenged with B16-F10 cells $1x10^5$ cells in right flank and $5x10^4$ cells in the left flank (a-b) or $1x10^5$ cells in the left flank (c-d) and were treated intratumorally with the indicated viruses and intraperitoneal anti-CTLA-4 antibody according to the schedule in figure 4b. a) Growth of individual treated and distant tumors with small tumor challenge and treatment with NDV-WT/anti-CTLA-4 combination. b) Overall survival with small tumor challenge and treatment with NDV-WT/anti-CTLA-4 combination. c) Growth of individual treated and distant tumors with larger tumor challenge and treatment with NDV-ICOSL/anti-CTLA-4 combination. d) Overall survival with small tumor challenge and treatment with NDV-ICOSL/anti-CTLA-4 combination. Pooled data from 2 experiments with 5 animals per group. ****p<0.0001.

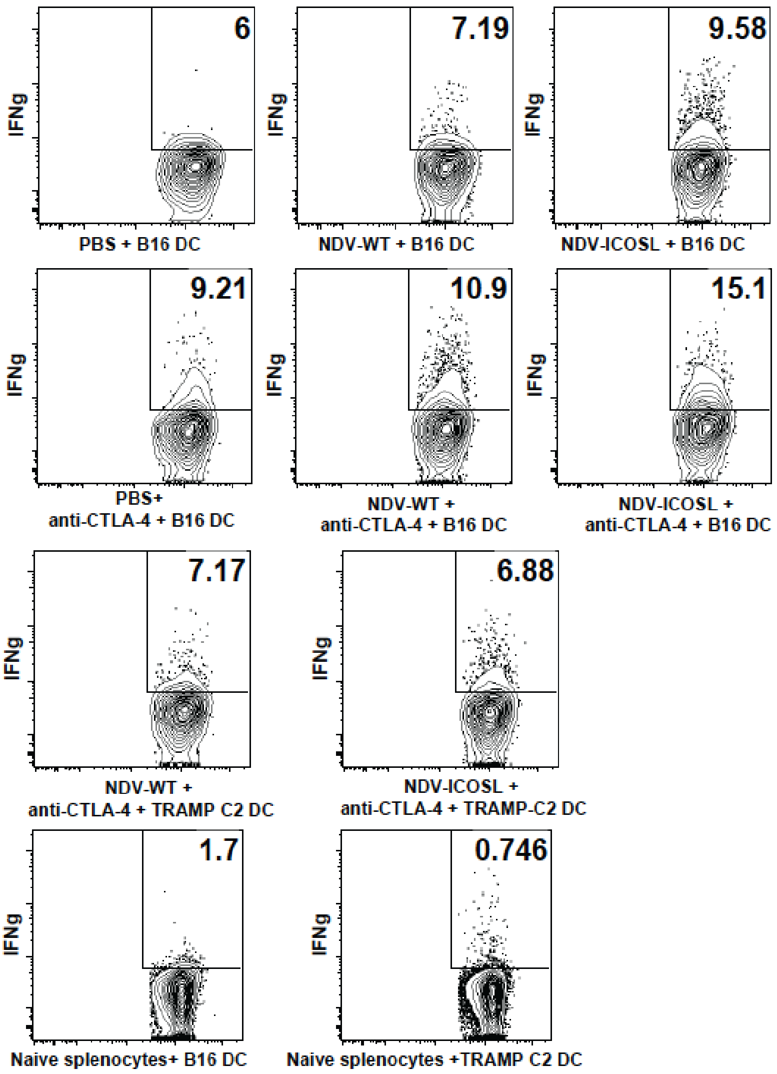

Supplementary Figure 8. Expression of IFNγ by tumor-infiltrating CD8+ lymphocytes in response to antigen. Splenic DC's loaded with B16-F10 or TRAMP-C2 tumor cell lysates were co-cultured with tumor T cells or naïve splenic T cells in a 1:5 (DC:lymphocyte) ratio for 8 hours. T cells were analyzed for IFNγ production by intracellular cytokine staining. Representative flow cytometry panels for each group and the controls are shown. Composite data for the analyses is shown in figure 5j.

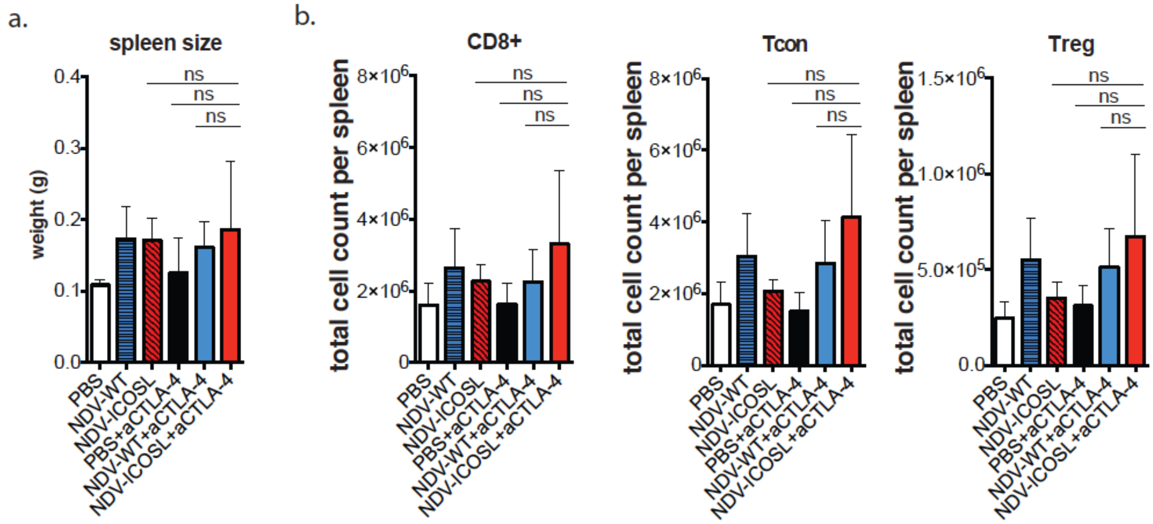

Supplementary Figure 9. Composition of splenic T cell populations.
Animals were treated according to the schedule in Figure 4b and spleens were collected on day 15. a) Spleen size in grams, b) Total calculated CD8+, Tcon, and Treg lymphocytes per gram of spleen. Data with error bars represent mean +/- SEM. Representative data from 2 experiments with 5 mice per group. ns: non-significant.

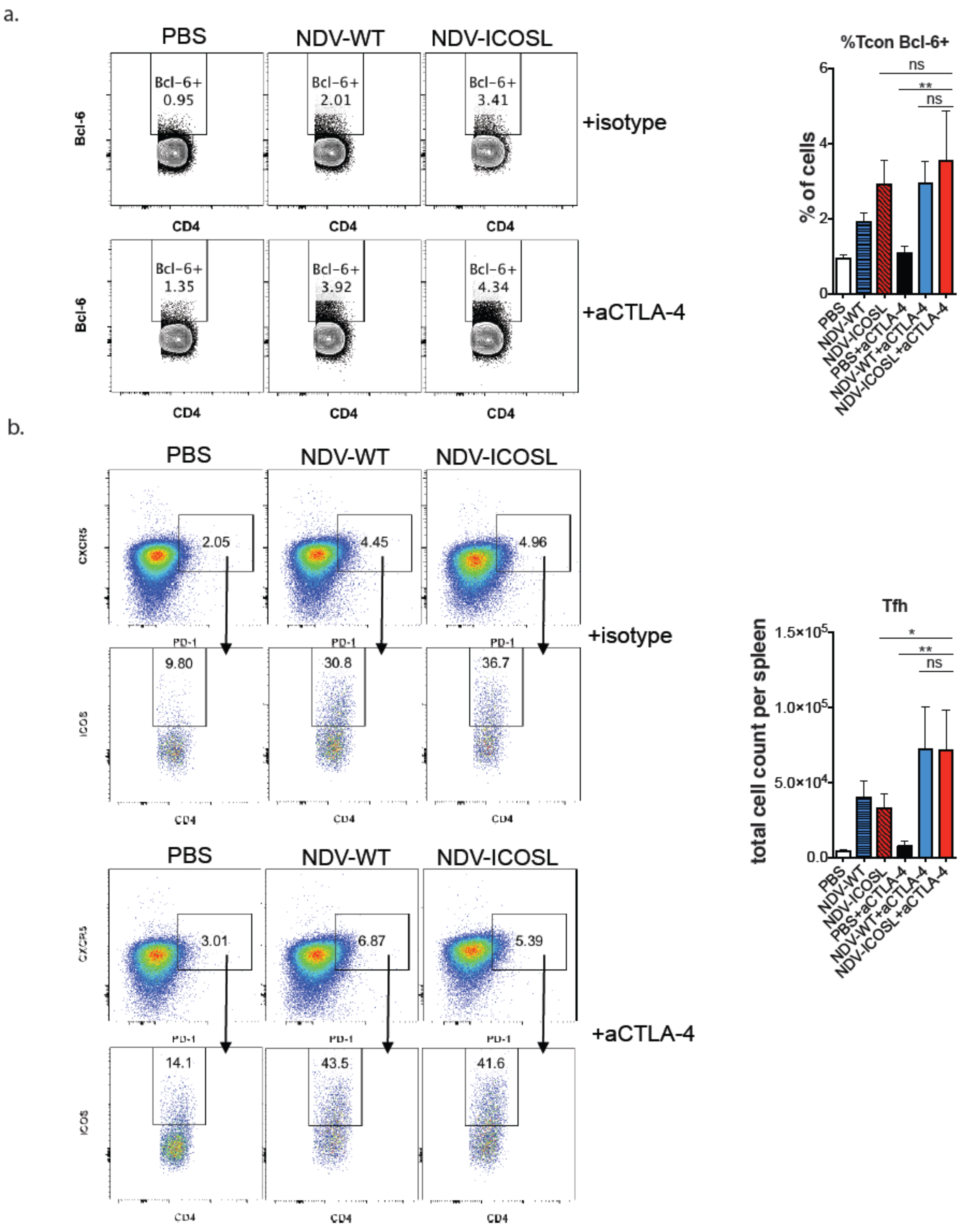

Supplementary Figure 10. Effect of NDV-ICOSL and CTLA-4 blockade on the splenic follicular helper T cells. Animals were treated according to the schedule specified in figure 4b and the spleens were collected on day 15. a) Expression of bcl-6 in CD4+FoxP3- (Tcon) subset. b) Overall percentages and gating strategy (left) and calculated absolute counts (right) of the splenic Tfh cells, as defined by $CD4^{+}FoxP3^{-}PD\text{-}1^{+}CXCR5^{+}ICOS^{+}$ lineage. Representative data of 2 experiments with 5 mice per group. Data with error bars represent mean +/- SEM. *p<0.05, **p<0.01, , ns: non-significant.

Supplementary Table 1. Upregulation of genes related to type I interferon.

Gene	Log2 fold change	Lower confidence limit	Upper confidence limit	P-value
Ifitm1	1.28	0.87	1.69	4.71E-06
Ifih1	0.367	0.239	0.494	1.40E-05
Cd3e	2.03	1.31	2.74	1.74E-05
Ifit2	0.807	0.502	1.11	3.92E-05
Ciita	1.13	0.695	1.56	4.61E-05
Ifi44	0.786	0.481	1.09	5.37E-05
Runx1	0.968	0.588	1.35	6.16E-05
Eomes	0.928	0.542	1.31	0.0001203
Tapbp	0.709	0.413	1.01	0.000125
Irf5	1.26	0.733	1.79	0.0001252
Icosl	0.708	0.411	1.01	0.0001308
Il12rb2	1.72	0.985	2.45	0.0001563
Gzmk	1.92	1.06	2.77	0.0002614
Sh2d1a	1.69	0.92	2.46	0.0003125
Gbp5	1.21	0.654	1.78	0.0003617
Nfkbia	1.08	0.578	1.58	0.0003878
Ifna1	1.1	0.58	1.61	0.0004463
Nod2	0.833	0.438	1.23	0.0004669
Irf8	0.982	0.489	1.47	0.0008162
H2.T23	0.846	0.418	1.27	0.0008728
Ifngr1	0.997	0.491	1.5	0.0008995
Tmed1	-0.177	-0.268	-0.0862	0.0009915
Irf7	0.581	0.271	0.891	0.001405
Ubc	0.211	0.0984	0.324	0.001413
Ifnar1	0.441	0.203	0.679	0.001546
Ifi44l	0.82	0.375	1.26	0.001629
H2.Aa	0.913	0.417	1.41	0.001661
Ifna2	0.953	0.422	1.48	0.002042
Ifnb1	0.785	0.34	1.23	0.002367
Ccr7	0.728	0.312	1.14	0.002516
Ifit3	0.404	0.172	0.635	0.002564
Masp2	0.932	0.394	1.47	0.002713
Cxcl16	0.963	0.397	1.53	0.003161
Ddx58	0.233	0.0742	0.391	0.009017
Ifna4	0.683	0.204	1.16	0.0109
Ifitm2	0.144	-0.0907	0.378	0.2429
Ifi27	0.0915	-0.0619	0.245	0.2554
Ifi35	-0.102	-0.311	0.106	0.3462

Fadd	0.0628	-0.0909	0.216	0.4323
Ifit1	0.0603	-0.176	0.297	0.6219

Supplementary Table 2. Upregulation of genes related to DC function

Gene	Log2 fold change	Lower confidence limit	Upper confidence limit	P-value
Ikzf2	0.891	0.658	1.12	2.37E-07
Ccr2	1.99	1.37	2.6	2.89E-06
Ly96	0.636	0.419	0.852	1.05E-05
Cd40	1.29	0.848	1.74	1.17E-05
Ccl5	1.94	1.21	2.67	3.90E-05
Cxcr1	1.07	0.662	1.48	4.29E-05
Cd40lg	1.23	0.75	1.7	5.38E-05
Ccr5	1.32	0.803	1.84	6.26E-05
Ccl19	1.62	0.969	2.27	8.11E-05
Cxcr4	1.12	0.646	1.59	0.0001414
Cr2	0.742	0.398	1.09	0.0003758
Ccr1	0.755	0.392	1.12	0.0005327
Cd86	1.19	0.568	1.81	0.001181
Rela	0.317	0.0828	0.552	0.01493
Cd83	0.492	-0.0137	0.999	0.07029
Tfrc	-0.239	-0.565	0.0876	0.1663

Supplementary Table 3. Upregulation of genes related to T cell function.

Gene	Log2 fold change	Lower confidence limit	Upper confidence limit	P-value
Cd2	2.15	1.64	2.67	5.40E-08
Ikzf2	0.891	0.658	1.12	2.37E-07
Cd4	2.05	1.51	2.6	2.95E-07
Tnfsf10	1.18	0.856	1.5	4.75E-07
Icos	2.57	1.83	3.3	8.76E-07
Il18r1	2.28	1.62	2.95	1.08E-06
Ctla4	1.82	1.29	2.34	1.10E-06
Cd5	1.91	1.35	2.46	1.15E-06
Btla	1.2	0.85	1.55	1.18E-06
Ifng	1.6	1.13	2.07	1.40E-06
Jak2	0.614	0.43	0.797	1.70E-06
Cd274	1.48	1.03	1.92	1.89E-06
Cd8a	2.14	1.49	2.79	2.10E-06
Itgal	1.88	1.31	2.45	2.17E-06
Gzmb	1.99	1.38	2.61	2.86E-06
Ccr2	1.99	1.37	2.6	2.89E-06
Il25	1.26	0.866	1.66	3.33E-06
Ccr3	1.29	0.88	1.69	3.81E-06
Stat4	1.36	0.931	1.8	3.92E-06
Itk	1.66	1.12	2.21	6.26E-06
Il12rb1	1.39	0.933	1.85	6.59E-06
Cd8b1	1.85	1.24	2.47	7.21E-06
Spn	1.87	1.24	2.49	7.88E-06
Il34	1.01	0.672	1.35	8.09E-06
Ccl11	2.02	1.34	2.69	9.02E-06
Il7r	2.1	1.39	2.81	9.04E-06
Tbx21	1.4	0.927	1.88	9.79E-06
Cd40	1.29	0.848	1.74	1.17E-05
Gata3	1.51	0.981	2.03	1.44E-05
Ptprc	1.94	1.26	2.63	1.65E-05
Thbd	0.964	0.622	1.3	1.71E-05
Cd3e	2.03	1.31	2.74	1.74E-05
Cd27	1.5	0.964	2.03	1.85E-05
Tlr9	1.38	0.887	1.88	2.08E-05
Cd3d	1.96	1.25	2.67	2.13E-05
H2.K1	0.606	0.387	0.824	2.21E-05
Itgam	1.3	0.825	1.77	2.42E-05
Ccl3	1.62	1.03	2.21	2.55E-05
Ripk2	0.434	0.275	0.593	2.69E-05

Cd247	1.68	1.06	2.3	2.95E-05
Cd3g	1.97	1.24	2.69	2.95E-05
Dpp4	1.65	1.03	2.27	3.47E-05
Cxcr3	1.04	0.653	1.44	3.58E-05
Ccl5	1.94	1.21	2.67	3.90E-05
Stat6	0.255	0.158	0.352	4.12E-05
Tank	0.306	0.189	0.423	4.44E-05
Fut7	1.11	0.685	1.54	4.52E-05
Ikzf1	1.69	1.04	2.34	4.64E-05
Twist1	1.05	0.646	1.45	4.68E-05
Psen1	0.208	0.128	0.289	4.85E-05
Tbk1	0.53	0.325	0.734	5.06E-05
Cd40lg	1.23	0.75	1.7	5.38E-05
Ccr6	1.14	0.695	1.59	5.66E-05
Ccr5	1.32	0.803	1.84	6.26E-05
Icam4	0.815	0.492	1.14	6.83E-05
Jak1	0.59	0.356	0.824	6.95E-05
Tnfrsf4	1.07	0.643	1.5	7.41E-05
Il2rb	1.81	1.09	2.54	7.59E-05
Bcl2	0.648	0.388	0.909	7.96E-05
Cd1d1	1.24	0.743	1.74	8.03E-05
Ccl19	1.62	0.969	2.27	8.11E-05
Irak4	0.261	0.156	0.367	8.67E-05
Gfi1	0.83	0.487	1.17	0.000111
Cd1d2	0.83	0.487	1.17	0.0001116
H2.D1	0.477	0.279	0.675	0.000115
Eomes	0.928	0.542	1.31	0.0001203
Klrk1	1.59	0.919	2.25	0.0001345
Cxcr4	1.12	0.646	1.59	0.0001414
Itch	0.467	0.269	0.664	0.0001449
Tnfsf15	1.14	0.658	1.63	0.0001464
Nfatc3	0.512	0.294	0.73	0.0001506
Il12rb2	1.72	0.985	2.45	0.0001563
Pou2af1	1	0.572	1.43	0.0001628
Maf	0.915	0.521	1.31	0.0001746
Traf3	0.46	0.262	0.658	0.0001762
Cd47	0.699	0.396	1	0.0001844
Stat3	0.388	0.218	0.558	0.000212
Il6ra	1.53	0.86	2.21	0.000214
Cd28	0.789	0.441	1.14	0.0002219
Il2	0.897	0.5	1.29	0.0002316

Gzmk	1.92	1.06	2.77	0.0002614
Lcp1	1.08	0.599	1.57	0.0002628
Il4ra	1.03	0.567	1.49	0.0002713
Cd48	1.34	0.739	1.94	0.0002724
Tnfrsf13b	1.03	0.568	1.5	0.0002768
Ccl2	0.877	0.479	1.27	0.0003034
Map3k5	0.508	0.276	0.74	0.0003193
Card11	1.42	0.772	2.08	0.0003306
Tnfsf13	0.81	0.437	1.18	0.0003516
Crebbp	0.804	0.433	1.17	0.0003588
Stat5b	0.385	0.207	0.563	0.0003619
Rag1	0.792	0.426	1.16	0.0003704
H2.M3	0.657	0.35	0.963	0.0004061
Pdcd1	1.71	0.912	2.51	0.0004117
Socs1	0.768	0.408	1.13	0.0004225
Nod2	0.833	0.438	1.23	0.0004669
Tnfrsf1b	1.71	0.897	2.52	0.0004744
Cd80	1.04	0.545	1.53	0.0004843
Ccl7	1.04	0.542	1.54	0.0005246
Il1rn	1.25	0.649	1.86	0.000557
Ncf4	1.26	0.652	1.87	0.0005753
Cd59b	0.58	0.299	0.862	0.000586
Tlr5	0.645	0.329	0.961	0.0006429
Snai1	1.03	0.522	1.54	0.0007037
Cma1	1.77	0.893	2.65	0.0007289
Csf2	0.981	0.493	1.47	0.0007527
Cxcl13	1.24	0.62	1.86	0.0007771
Bcl10	0.372	0.185	0.558	0.0008024
Il7	0.576	0.287	0.866	0.0008223
Tnfsf18	0.589	0.292	0.885	0.0008486
Ccr4	0.944	0.468	1.42	0.0008491
Fcgr4	0.967	0.478	1.46	0.000867
Il6st	0.737	0.364	1.11	0.0008773
Pax5	1.02	0.495	1.54	0.001026
Ythdf2	0.219	0.106	0.332	0.001053
Tlr3	0.463	0.223	0.703	0.001103
Cd86	1.19	0.568	1.81	0.001181
Ifnar2	0.39	0.185	0.595	0.001242
Il4	0.901	0.427	1.37	0.001255
Fas	1.01	0.479	1.55	0.001257
Nfatc1	1.25	0.588	1.91	0.001303

Irf7	0.581	0.271	0.891	0.001405
Foxp3	0.935	0.436	1.43	0.001412
Ido1	0.654	0.303	1.01	0.00149
H2.Aa	0.913	0.417	1.41	0.001661
Fasl	1.04	0.474	1.61	0.001708
Relb	0.634	0.286	0.981	0.001793
H2.Eb1	0.982	0.443	1.52	0.001815
Txnip	0.752	0.336	1.17	0.001921
Ccr7	0.728	0.312	1.14	0.002516
Tollip	0.252	0.108	0.395	0.002524
Prkce	0.372	0.154	0.589	0.00308
Rora	0.615	0.254	0.976	0.003126
H2.Ab1	0.88	0.353	1.41	0.003625
Bcl6	0.633	0.253	1.01	0.003722
Il17rb	0.838	0.329	1.35	0.004054
Tnfsf13b	0.829	0.325	1.33	0.004071
Ctsh	0.943	0.368	1.52	0.004174
Map4k2	0.64	0.25	1.03	0.004183
Il17a	0.789	0.305	1.27	0.004322
Cebpb	0.783	0.301	1.26	0.004488
Tgfb1	0.702	0.267	1.14	0.004701
Cd74	0.771	0.29	1.25	0.004936
Foxj1	0.665	0.241	1.09	0.005733
Itgae	0.496	0.179	0.814	0.005907
Il1b	0.837	0.3	1.37	0.006014
Sele	0.645	0.23	1.06	0.00615
Il11ra1	0.688	0.244	1.13	0.006239
Il18	0.235	0.0825	0.387	0.006512
H2.Q2	0.584	0.205	0.964	0.006552
Tnfrsf13c	0.787	0.273	1.3	0.006756
Ccnd3	0.229	0.0781	0.38	0.007269
Tmem173	0.351	0.118	0.584	0.007603
Ifna4	0.683	0.204	1.16	0.0109
Casp3	0.226	0.0638	0.389	0.01259
Ikbkg	0.262	0.0729	0.451	0.01295
Flt3	0.659	0.177	1.14	0.01396
Il12b	0.58	0.155	1.01	0.01417
Rela	0.317	0.0828	0.552	0.01493
Il13	0.526	0.132	0.92	0.01612
Hamp	0.564	0.141	0.986	0.01624
Trem2	0.797	0.197	1.4	0.01666

St6gal1	0.315	0.0721	0.559	0.01904
Cxcl12	0.947	0.199	1.69	0.0216
Itgb1	0.183	0.0318	0.335	0.02738
Mif	-0.195	-0.359	-0.0314	0.02952
Tnfsf4	0.367	0.0588	0.676	0.02964
Isg20	0.354	0.0345	0.673	0.04145
Ptgs2	0.777	0.0602	1.49	0.04567
Yy1	0.122	0.00846	0.236	0.04741
Lbp	0.627	-0.00449	1.26	0.06515
Cd276	0.164	-0.00384	0.331	0.0692
Cd83	0.492	-0.0137	0.999	0.07029
Vcam1	0.532	-0.018	1.08	0.07181
Usp18	0.194	-0.0129	0.4	0.08031
Egr1	0.378	-0.041	0.797	0.09152
Lcn2	-0.457	-1.06	0.143	0.1502
Gpr44	0.397	-0.145	0.938	0.1659
Tfrc	-0.239	-0.565	0.0876	0.1663
Irf3	0.0944	-0.0406	0.229	0.1848
Xbp1	-0.102	-0.249	0.0454	0.1897
Hspb2	-0.153	-0.389	0.0821	0.2156
Anxa1	0.118	-0.0957	0.332	0.2915
Il1a	0.193	-0.169	0.555	0.3078
Tie1	0.159	-0.139	0.456	0.3079
Itgb4	0.229	-0.203	0.662	0.3107
Psma2	0.0486	-0.0502	0.147	0.346
Pvr	0.0809	-0.0845	0.246	0.3486
Tgfb2	0.096	-0.107	0.299	0.3638
Il22ra2	-0.203	-0.654	0.247	0.3866
Ada	-0.0681	-0.232	0.0961	0.4253
Il5	0.123	-0.313	0.558	0.5868
Il12a	0.0683	-0.255	0.392	0.6836
Rorc	0.0111	-0.175	0.197	0.9082
Mapk3	-0.0039	-0.156	0.148	0.9605
